# Improved Functionality, Quality, and Shelf Life of *Merguez*-Type Camel Sausage Fortified with Spirulina as a Natural Ingredient

**DOI:** 10.3390/foods14010059

**Published:** 2024-12-28

**Authors:** Djamel Djenane, Boumediène Méghit Khaled, Yamina Ben Miri, Mohammed Said Metahri, Luis Montañés, Mohammed Aider, Agustín Ariño

**Affiliations:** 1 Meat Quality and Meat Safety Laboratory, University Mouloud Mammeri, Tizi Ouzou 15000, Algeria; sys_yamina@yahoo.com; 2 Laboratoire de Nutrition, Pathologie, Agro-Biotechnologie et Santé (Lab-NuPABS), Department of Biology, Faculty of Natural Sciences and Life, Djillali Liabès University, Sidi Bel Abbès 22005, Algeria; khaledmb73@gmail.com; 3 Department of Biochemistry and Microbiology, Faculty of Sciences, Mohamed Boudiaf University, M’sila 28000, Algeria; 4 Faculty of Biological and Agricultural Sciences, Mouloud Mammeri University of Tizi-Ouzou, Tizi Ouzou 15000, Algeria; metahriummtodz@gmail.com; 5 Laboratorios Valero Analítica S.L., 50011 Zaragoza, Spain; lmontane@valeroanalitica.com; 6 Department of Soil Sciences and Agri-Food Engineering, Université Laval, Quebec City, QC G1V 0A6, Canada; mohammed.aider@fsaa.ulaval.ca; 7 Institute of Nutrition and Functional Foods (INAF), Université Laval, Quebec City, QC G1V 0A6, Canada; 8 Facultad de Veterinaria, Instituto Agroalimentario de Aragón-IA2, Universidad de Zaragoza-CITA, 50013 Zaragoza, Spain; aarino@unizar.es

**Keywords:** spirulina, camel meat, sausages, vacuum storage, quality attributes, prebiotic, shelf-life, foodborne pathogens

## Abstract

The objective of the present work was to examine the effect of incorporating spirulina powder (SP) in *merguez*-type sausages made exclusively with camel meat, as well as to evaluate its physicochemical, microbiological, and sensory quality attributes and its prebiotic potential. The final purpose was to offer an innovative meat product to increase camel meat consumption. Several innovative fresh sausage formulations were developed using SP (00, 100, 250, and 500 mg/kg) and stored under vacuum conditions with refrigeration at 1 ± 1 °C for 35 days. A control group of camel sausage without SP was also stored overwrapped (OW) under aerobic conditions, to serve as the negative control. The addition of SP to the vacuum-packed camel sausages extended their shelf life by 20 to 35 days compared to the control group, which was completely spoiled by the fifth day of storage. These results were more pronounced the higher the percentage of SP incorporated into the camel sausage formulation, as indicated by the following parameters: 2-thiobarbituric acid-reactive substances TBARS (1.46 vs. 2.89 mg MDA/kg), CIE a* (14.65 vs. 10.12), total volatile basic nitrogen TVB-N (13.02 vs. 15.09 mg/kg), total psychrotrophic bacteria TPB (5.71 vs. 6.34 log CFU/g), and overall acceptability score (3.17 vs. 2.5). The study of prebiotic potential suggested that the addition of SP to camel sausages promoted the growth of probiotic strains, which in turn were able to inhibit the growth of pathogenic microorganisms such as *S. aureus* and *E. coli* O157:H7. In conclusion, this study highlighted how SP, as a clean label ingredient, based on its rich composition and its antioxidant, antibacterial, and prebiotic effects, may represent a source of beneficial substances for human health and offer an alternative approach to producing a new traditional *merguez*-type sausage with improved acceptance.

## 1. Introduction

The United Nations has designated 2024 the year of the camel. Consequently, extensive camel breeding for meat and milk has expanded in several countries, particularly Algeria. Camel (*Camelus dromedarius*) meat possesses recognized nutritional properties compared to other meats such as beef and mutton [1]. Furthermore, there is growing consumer demand for nutritious, healthy, and easy-to-prepare products. Given this context, it may be worthwhile developing novel camel-based products that fulfil these requirements while being attractive in terms of quality and affordability. However, the introduction of camel meat into consumers’ diets presents technological challenges, primarily due to its rigidity attributed to higher connective tissue [1,2], as camel meat frequently comes from older animals. This high amount of connective tissue makes camel meat a challenging raw material for producing acceptable products. Camel meat derivatives, particularly fresh sausages (*merguez*-type), represent a promising option to address these challenges, as they hold significant cultural value in Algeria and have the potential to achieve a substantial market position within the processed meat sector.

In several regions of Algeria, particularly in semi-arid and desert areas, camel meat is still sold fresh under poor hygienic conditions: it is typically offered as small, unprotected, fresh pieces. It is well established that camel meat is highly susceptible to rapid spoilage when stored improperly, making it a potential source of foodborne infections [3]. The majority of meat derivatives currently marketed contain synthetic ingredients such as additives and an excess of other components such as salt and saturated fat, the consumption of which should be moderated. However, consumers are becoming increasingly aware of the relationship between the food they consume and their health. The development of a healthier fresh camel sausage of the *merguez* type presents a promising alternative for the desert-dwelling populations of Algeria. The meat industry should be aware of the demands and interests of consumers, encouraging the development of new meat products that meet the criteria of being safe, nutritious, and healthy.

According to recent research conducted within the framework of the “Sustainable Development Goals”, spirulina, a cyanobacterium, has emerged as a promising source of protein and other bioactive molecules. Spirulina contains healthy nutrients such as essential amino acids, minerals (especially iron), omega-3 polyunsaturated fatty acids (PUFAs), antioxidants, and vitamins, as well as other compounds (pigments such as chlorophylls, carotenoids, phycocyanin, and polyphenolic compounds). Recently, spirulina has shown strong prebiotic potential to enhance the antibacterial effect of probiotics against pathogens [4]. In addition, spirulina stands out as a sustainable food source, offering minimal water and carbon footprints and less need for arable land compared to traditional crops [5,6]. For all these reasons, several innovative/novel food products incorporating spirulina have been developed by the food industry, including fermented dairy beverages [7,8], mayonnaise [9], soy-yogurt biscuits [10], ice cream [11], and gluten-free pasta and bread for celiac patients [12], among others. Spirulina has also been incorporated into packaging films to produce bioactive and intelligent industrial food packaging films [13,14]. Beyond human food, spirulina’s application as a feed supplement has been investigated for its effect on animal performance and animal product quality across various species, such as buffaloes [15], broilers [16], and ruminants [17]. However, there have been no reports to date regarding the addition of spirulina to camel-based products.

Fresh sausages are generally stored in open air or simply overwrapped without adequate protection. Furthermore, fresh minced meat provides a favorable background for the growth of pathogens, including *Escherichia coli* O157:H7 and *Staphylococcus aureus*, that are common in camels’ pastoral environment [18], and are responsible for food poisoning concerns. To guarantee the quality and safety of meat, vacuum packaging (VP), modified atmosphere packaging (MAP), and active packaging, either alone or in combination with natural preservatives, have proven effective in substantially extending the shelf life of meat products [19]. In parallel, changes in consumer lifestyles have made the demand for high quality, minimally processed, ready-to-cook sausages with a longer shelf life and typical traditional flavor a major challenge for the meat industry. In light of the growing interest in spirulina cyanobacteria and their potential uses, the aim of this study was to assess the functionality, quality, and shelf life of *merguez*-type camel sausage fortified with spirulina as a natural ingredient. To understand the beneficial effects of spirulina addition, antioxidant capacity, antimicrobial activity, and efficacy as a prebiotic agent were evaluated, with the ultimate goal of producing a new camel *merguez*-type sausage. The quality of the final product stored under vacuum conditions for 35 days was evaluated in terms of functionality, physicochemical properties, and microbiological properties, compared to a standard formulation (without spirulina addition). Additionally, this study examined the effect of spirulina enrichment on the sensory profile and shelf life of the product during storage. This work may encourage meat processors to use camel meat to produce high-quality meat products.

## 2. Materials and Methods

### 2.1. Ingredients and Chemical Reagents

Camel meat and humped fat obtained from a local butcher (Boucherie Hanafi, Bordj-Ménaïel, Algeria) were used in this study. Spirulina powder (SP) (powdered cyanobacteria biomass *Arthospira platensis* spp.) was purchased from Spirulina Algérie, Sarl, Algeria. The ingredients for the *merguez* spices mixture (dry fine powder), including garlic, caraway, black pepper, coriander, hot pepper, and paprika, as well as pasteurized whole liquid eggs and salt, were purchased from local markets. The spice powder was packed in an individual polypropylene bag with an aluminum coating and stored in cool and dry conditions, away from sunlight, until further processing of the artisanal Algerian *merguez*-type camel sausages (hereafter referred to as camel sausages). Before use, the SP was dissolved in sterile distilled water, and the suspension was sonicated for 2 min.

All reagents used for chemical analysis were purchased from Chetouane Ets. (Boumerdes, Algeria) and were certified as 100% pure and of analytical quality.

### 2.2. Experimental Design

The camel meat used in this trial was handled in accordance with the guidelines established by the Algerian Ministry of Agriculture (JORA No. 65 of 30 October 1996; Order of 15 July 1996, specifying the characteristics and procedures for the labeling of butcher’s meat).

**Figure 1:**
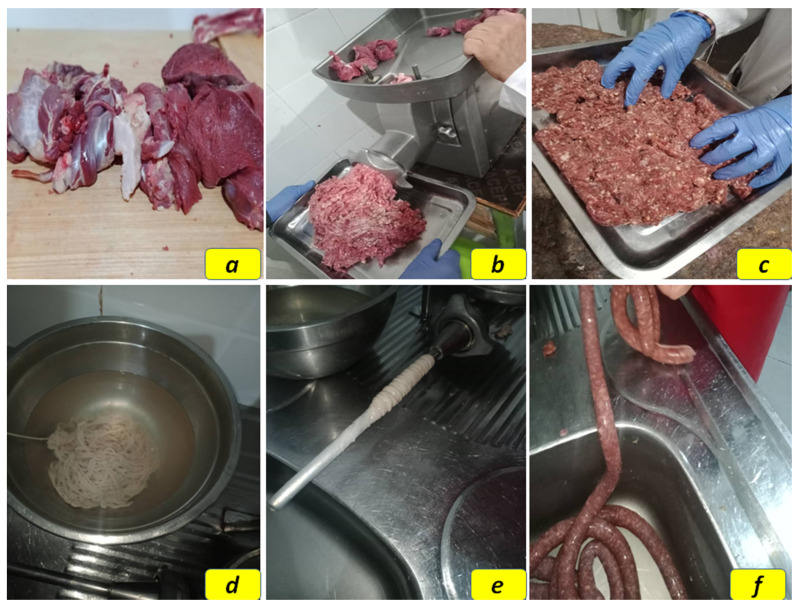
The process of preparing artisanal Algerian *merguez*-type camel sausages: (**a**) preparation of camel meat and fat; (**b**) mincing meat and fat together with spirulina powder; (**c**) addition of *merguez* spices mixture and salt; (**d**) preparation and cleaning of fresh sheep casings; (**e**) casing preparation and set-up before filling; (**f**) artisanal *merguez*-type camel sausages.

The experimental design is shown in Figure 1. A three replicate-based experiment (three independent replicates at different times) was carried out to investigate the effect of incorporating SP during the formulation of camel sausages on the physicochemical, microbiological, and sensory characteristics of the manufactured product. Therefore, four camel sausage formulations were prepared: (1) control sausages without spirulina (SP-00), (2) sausages with 0.01% spirulina (SP-100, 100 mg/kg), (3) sausages with 0.025% spirulina (SP-250, 250 mg/kg), and (4) sausages with 0.05% spirulina (SP-500, 500 mg/kg). All four groups were held in vacuum storage, and a fifth group (OW) was made up of negative control sausages (without spirulina) but stored overwrapped under aerobic conditions. For vacuum storage, the sausages were individually placed in plastic bags with high barrier properties, vacuum packaged, and stored in darkness at 1 ± 1 °C for 35 days. The samples were examined at different time intervals during storage (0, 5, 10, 15, 20, 25, 30, and 35 days) for pH, total volatile basic nitrogen (TVB-N), instrumental color measurements (CIE L*a*b* system), 2-thiobarbituric acid reactive substances (TBARS), and cooking characteristics, as well as microbiological and sensory qualities.

### 2.3. Preparation of Merguez-Type Camel Sausages

A preliminary camel sausage-manufacturing test was conducted in the laboratory to determine the appropriate proportions of the various ingredients and the appropriate levels of SP enrichment. Finally, SP concentrations of 0% (control), 0.01%, 0.025%, and 0.05% (*w*/*w*) were selected for further testing. These SP concentrations were selected considering the organoleptic characteristics of the final product tailored to the preferences of the local market. Note that a fifth group (OW) comprised control sausages (without spirulina) stored overwrapped under aerobic conditions. For the preparation of camel sausages, fresh boneless *Biceps femoris* muscles obtained from 7- to 8-year-old camels (*Camelus dromedarius*) were cut into small cubes (~2 × 2 × 2 cm^3^) after removing visible fat and connective tissues. Camel meat and camel hump fat were ground separately through a 3 mm plate grinder (Cgt C22/300, Equipro Sarl., Béjaia, Algeria). The minced camel meat (70%) and fat (20%) were thoroughly mixed with SP, pasteurized whole liquid eggs, and a traditional *merguez* spice mixture containing black pepper, garlic, caraway, coriander, hot pepper, paprika, and salt, according to the proportions shown in Table 1.

The sausage formulations were prepared as described above. Each sausage type was formulated to be approximately 70% lean meat and 20% fat and was stuffed into 10 mm ø natural sausage casings made from clean sheep intestines and cut into 10 cm long pieces. Then, the obtained camel sausage formulations were placed on plastic foam trays, vacuum-packed in transparent multilayer PA/PE bags (low-density polyethylene (LD-PE/Polyamide (PA) coextruded film using a vacuum packing machine (VAC-20 SL 2A, EDESA, Barcelona, Spain), and stored in the dark at 1 ± 1 °C for 35 days. Additionally, a separate batch of control sausages (without spirulina) was prepared but stored overwrapped under aerobic conditions. As previously noted, all samples were examined at specific time intervals during storage (0, 5, 10, 15, 20, 25, 30, and 35 days).

### 2.4. Chemical Analysis of the Spirulina Powder and Camel Sausages

For chemical composition analysis of the SP, samples were directly taken from the original packages. For chemical analysis of the experimental camel sausages, freshly prepared samples were cut into pieces and homogenized using a grinder (Ultra Turrax, IKA works Inc., Staufen im Breisgau, Germany). Crude protein, lipid content, total carbohydrates, moisture, and ash were determined following the methods of the Association of Official Analytical Chemists (AOAC) [20]. Briefly, crude protein content was determined using the Kjeldahl method, using conversion factors of 5.95 for SP and 6.25 for camel sausages. The Soxhlet extraction method was employed to determine the level of crude fat, with petroleum ether (boiling point, 40–60 °C) as the solvent. Ash content was obtained following incineration at 550 °C for 4–6 h in a muffle furnace. Moisture content was determined based on weight loss of the sample during thermal drying at 103 ± 2 °C until a constant weight was achieved. Total carbohydrates were calculated by determining the residual weight after subtracting the water, protein, fat, and ash amounts found by analysis. Finally, energy values for 100 g (kcal and kJ) were calculated using the conversion factors specified in EU Regulation No 1169/2011 (4 kcal/g and 17 kJ/g for protein and carbohydrates, 9 kcal/g and 37 kJ/g for fat) [21,22,23].

### 2.5. Total Phenolic Compounds in the Spirulina Powder

Total phenolic compounds (TPC) in the SP were determined according to the Folin–Ciocalteu method [24], with slight modifications. Two and a half milliliter (2.5 mL) portions of the Folin–Ciocalteu reagent were added to aliquots of 0.5 mL of aqueous-methanolic extract, and the mixture was incubated in the dark for 10 min (reaction). Subsequently, 2 mL of a sodium carbonate solution (75 g/L) was added, and the mixture was kept in the dark for 45 min. The assay tubes were vortexed and incubated at 45 °C for 10 min, then cooled. For the blank assay, 0.5 mL of distilled water was used. The mixture absorbance was then measured at 760 nm using a Jasco V-360 spectrophotometer (Jasco, Tokyo, Japan). Gallic acid was used as phenolic compound standard for constructing the calibration curve, and the TPC of spirulina powder was expressed as milligrams of gallic acid equivalents per gram of sample dry weight (mg GAE/g). Data are presented as the average of triplicate analyses.

### 2.6. Determination of the pH of Camel Sausages

The pH of the camel sausages was determined with a digital puncture pHmeter (XS Instruments, Mod. PH25, Carpi, MO, Italy) by inserting the electrode directly into the samples. Previously, the instrument was calibrated with buffers of pH 4.01 and 7.00 (XS Instruments).

### 2.7. Oxidative Stability Analysis (TBARS) of Camel Sausages

In order to determine the extent of lipid oxidation of camel sausages during storage, the 2-thiobarbituric acid reactive substances (TBARS) method was conducted, as described by Djenane et al. [25]. As a brief description, 20 g of sausage was blended with 20 mL of trichloroacetic acid (TCA: 20% *w*/*v*). The mixture was centrifuged at 14,000 rpm for 60 s in an ice bath, after which 2 mL of supernatant was collected from each sample. The collected supernatant was filtered (Whatman grade 42 circles, GE Healthcare Life Sciences, Gillingham, UK), and 2 mL of 20 mM TBA aqueous solution was added to the filtrate. The resulting mixture was incubated at 90 °C for 60 min in a water bath. After cooling the samples to room temperature, the absorbance was measured at 531 nm using a UV-1800 spectrophotometer (Shimadzu, Tokyo, Japan). All measurements were compared with a control sample that did not contain sausage. A standard curve generated using 1,1,3,3-tetraethoxypropane was used to calculate the amount of malondialdehyde (MDA) produced. Three replicate measurements were performed for each sample, and the mean value ± standard deviation (SD) is reported. Results are expressed as mg of MDA equivalents/kg sausage.

### 2.8. Microbiological Analysis of Camel Sausages

For the microbiological analysis of camel sausages during storage, tenfold dilution series were prepared in saline (0.85%) peptone (0.1%) water for plating. An inoculum of 1 mL from each dilution was plated, in triplicate, onto the appropriate agar for each microbial species. Total psychrotrophic bacteria (TPB) were enumerated on Plate Count Agar (PCA; Merck, Darmstadt, Germany) incubated at 7 °C for 10 days. *Brochothrix thermosphacta* was enumerated on streptomycin thallous acetate actidione (STAA) agar (Biolife s.r.l; Milano, Italy) incubated aerobically at 25 °C for 72 h. Lactic acid bacteria (LAB) were enumerated on De Man, Rogosa, and Sharpe agar (MRS; Oxoid, Madrid, Spain) incubated anaerobically at 30 °C for 48–72 h. The results are expressed as log CFU/gram.

### 2.9. Instrumental Color Measurement of Camel Sausages

The color of camel sausage was measured instantly after opening the package; no blooming time was allowed, to ensure that the color was recorded as close to consumers’ perception through the packaging during retail sale. The color values—CIE L* (lightness; 0 = black, 100 = white), CIE a* (redness/greenness; positive value = red, negative values = green), and CIE b* (yellowness/blueness; positive values = yellow, negative values = blue)—were determined according to CIE (Commission Internationale de l’Eclairage) and were taken on the surface of the raw samples using a Minolta CR-400 Chroma Meter (Konica Minolta, Chiyoda, Tokyo, Japan) calibrated against black and white plate standards, with a 10 mm diameter measuring area and a D65 illuminant, as described by Camo et al. [26].

### 2.10. Determination of Total Volatile Basic Nitrogen (TVB-N) in Camel Sausages

TVB-N is frequently used as a biomarker of animal product spoilage because of its positive involvement in both microbiological growth and the proteolytic enzyme activity responsible for meat spoilage. The progression of protein degradation in camel sausages was evaluated through TVB-N determination and was measured by semi-micro steam distillation, as described by Djenane et al. [19].

### 2.11. Cooking Characteristics of Camel Sausages

Camel sausages were roasted in a preheated electric grill (Jata GR217, Madrid, Spain) at around 180 °C for 3 min, during which the temperature in the center of the samples reached approximately 80 °C. Cooking measurements were carried out on three replicates for each treatment. The difference in weight of samples before and after cooking was expressed as a percentage of cooking loss.

Cooking loss = [Raw sample weight − Cooked sample weight]/[Raw sample weight] × 100

Width reduction (%) = [Raw sample width − Cooked sample width]/[Raw sample width] × 100

Length reduction (%) = [Raw sample length − Cooked sample length]/[Raw sample length] × 100

Shrinkage (%) of camel sausages was calculated using the following Equation:

[(Raw sample length − Cooked sample length) + (Raw sample width − Cooked sample width)]/[Raw sample length + Raw sample width] × 100

### 2.12. Sensory Analysis of Camel Sausages

Sensory analysis of the camel sausages focused on assessing odor intensity and overall acceptability using a five-point system by a trained panel of six food technologists, according to the method described by Djenane et al. [2]. The panelists were selected based on their previous experience and expertise with sensory analysis of meat products. The sensory sessions took place at the laboratory of Meat Quality and Safety Research (University of Tizi-Ouzou, Tizi Ouzou, Algeria), under controlled conditions, including white lighting and at a room temperature of 25 ± 1 °C. The samples were served alone, without condiments or additional ingredients, to ensure unbiased evaluation. Odor intensity was based on a five-point scale, where 5 = none and 0 = poor. For overall acceptability, the scale was 0 = dislike very much to 5 = like very much. The camel sausages were cooked exactly as described for cooking loss above.

### 2.13. Prebiotic Effects of Spirulina Powder on Probiotic Cultures and Its Antimicrobial Activity

The probiotic strains used in this work were *Lactobacillus salivarius* CECT 4063 and *Pediococcus acidilactici* CECT 9879, while the foodborne pathogens used were *Staphylococcus aureus* CECT 4459 and *Escherichia coli* O157:H7 CECT 4267. All strains were provided by the Spanish Type Culture Collection (CECT) and stored in cryovials (Deltalab, Barcelona, Spain: Ref. 409113/6) at −80 °C. To standardize the inoculum, each probiotic strain was incubated in Man, Rogosa, and Sharpe (MRS) broth (Merck; Darmstadt, Germany) at 37 °C for 24 h in an anaerobic jar (Anaero Jar TM 2.5l, Oxoid Ltd., Basingstoke, UK) containing a gas-generating package (AnaeroPack, Mitsubishi Gas Chemical Co., Tokyo, Japan). The foodborne pathogen strains were incubated in brain heart infusion (BHI) broth (Oxoid Ltd. Ref: CM0225) at 37 °C for 24 h. After incubation, their concentrations were measured by spectrophotometry (Spectronic 20, Bausch and Lomb, Rochester, NY, USA) and confirmed by plating on plate count agar (PCA). The final bacterial loads were 1.5 × 10^9^ CFU/mL for *Lactobacillus salivarius*, 3.2 × 10^9^ CFU/mL for *Pediococcus acidilactici*, 1.2 × 10^8^ CFU/mL for *Staphylococcus aureus*, and 2.5 × 10^8^ CFU/mL for *Escherichia coli* O157:H7.

The prebiotic impact of SP on both probiotic strains was tested in flat-bottom 96-well plates incubated at 37 °C for 24 h on an orbital continuous shaker set at 150 rpm. For this, serial twofold dilutions were made in a concentration range of spirulina from 0.02% to 10% (*v*/*v*) in 10 mL sterile test tubes containing pure probiotic cultures in MRS broth. Bacterial growth was monitored by measurement of the optical density (OD at 630 nm) at 3 h intervals, for a total period of 24 h.

The ability of the selected probiotic strains to inhibit the growth of pathogenic bacteria was evaluated in co-cultures using the plate count method. Overnight cultures of *S. aureus* and *E. coli* were suspended in tryptic soy broth with yeast extract (TSB-YE), buffered with 50 mM sodium phosphate to pH 5.6–5.8, to simulate the normal pH of meat. Fresh camel sausage samples (5 g) containing SP were stirred in flasks containing 50 mL of 0.1% sterile peptone water to produce meat broth. One milliliter of each pathogenic bacterial suspension and one milliliter of each probiotic suspension was added successively to each flask. The flasks containing the mixture of camel sausage broth supplemented with SP, probiotic, and pathogen were incubated at 8 ± 2 °C for 8 days to stimulate typical supermarket refrigeration conditions. Changes in the *S. aureus* count were evaluated by plating on Baird–Parker agar (Oxoid; CM275) supplemented with egg yolk–tellurite emulsion (Oxoid; SR054C) and incubated at 37 °C for 48 h. *E. coli* were enumerated on Cefixime-Tellurite Sorbitol MacConkey (CT-SMAC) agar (DIFCO Lab, Detroit, MI, USA) incubated at 37 °C for 24 h. The analysis included the impact of all categorical variables, including probiotic strain, prebiotic SP, and SP concentration, as well as the pathogen strain, for which each sample was assessed in triplicate and counts are expressed as log CFU/mL.

### 2.14. Statistical Data Analysis

All experiments were conducted in triplicate for each treatment, and the values are presented as the mean ± standard deviation (SD). Analysis of variance (ANOVA, one-way analysis) was conducted, and the data were subjected to post hoc analysis using Duncan’s test for determining significance at a *p* < 0.05 level using Statistical Package for the Social Sciences software (SPSS version 21, IBM Corporation, Armonk, NY, USA).

## 3. Results and Discussion

### 3.1. Chemical Composition and Total Phenolic Compounds (TPCs) of Spirulina Powder

Table 2 shows the chemical composition of the spirulina powder used in this study. Analysis of the SP showed a higher protein content (66.88%), as well as a high percentage of carbohydrates (20.73%) and ash (5.11%) and a low fat content (3.13%), which contributes to its relatively low caloric value (378 kcal/100 g). These values are in agreement with previously published work [15,27,28,29]. The potential health benefit of spirulina is mainly due to its chemical composition, which includes essential amino acids, proteins, minerals (especially iron), and high concentrations of omega-3 PUFAs and vitamins [15,30,31,32,33]. Due to its high protein content, SP is one of the richest sources of protein among plant-based materials or animal protein sources. Therefore, the incorporation of these algae into meat products would improve their content of all the nutrients mentioned. Proteins, in particular, play a crucial role not only in boosting nutritional value but also in improving the texture and cooking quality of sausages. As a result, camel sausages enriched with SP can be considered a healthier alternative to the conventional sausages currently available on the market. Due to its high protein content, spirulina is widely employed as an additive in various food products [32,34,35]. For instance, pasta enriched with spirulina (1.5%) showed significant improvements in nutritional values compared to conventional whole-grain pasta [27,34]. Table 2 presents also the results of total phenolic compounds (TPCs) in SP that attained a concentration of 8.94 ± 0.16 mg GAE/g sample. Phenolic compounds are considered an important source of bioactive molecules with antimicrobial and antioxidant activities.

Apak et al. [36] reported a spirulina with a TPC content (26.64 mg GAE/g) approximately three times higher than that found in our work (8.94 mg GAE/g) and demonstrated its ability to inhibit 46% of free radicals. In contrast, Matos et al. [37] found lower concentrations of polyphenols in alcoholic (2.05 mg GAE/g) and aqueous (3.34 mg GAE/g) extracts of spirulina. Some of the phenolic compounds that can be found in spirulina extracts are catechin, epicatechin, pyrocatechol, chlorogenic and vanillic acids, caffeine, hesperidin, gallic acid, naringenin, naringin, pyrogallol, p-coumaric acid, quercetin, and rutin [36,37]. The antioxidant activity of spirulina extract may also be affected by the content of natural pigments such as chlorophylls, carotenoids, allophycocyanin, phycoerythrin, and phycocyanin [38]. For example, spirulina phycocyanin exhibited dose-dependent antioxidant activity in vitro [39], as did spirulina extracts that largely scavenged DPPH radicals in a dose-dependent manner [40]. Other studies found that spirulina extract contained variable amounts of TPC, with values ranging from 3.5 mg GAE/g in ethanolic extract to 9.1 mg GAE/g in aqueous extract [41] and from 2.12 mg GAE/g in fresh matter to 6.92 mg GAE/g in dry matter [42]. Furthermore, it was reported that spirulina extract containing between 4.50 and 27 mg GAE/g was able to inhibit 42–50% of free radicals [29,41].

Rose et al. [28] found that variations in the percentage of DPPH inhibition and total phenolic contents in spirulina might be attributed to different stages of algal maturity and harvesting, as well as processing, such as drying and milling operations [28]. Agustini et al. [42] observed that a spirulina whose phenolic content ranging between 2.12 and 6.92 mg GAE/g achieved an IC50 value of around 33 for DPPH scavenging activity. The presence of several factors explains the wide variations in the TPC values of spirulina reported in the literature (algal species, origin, growth conditions, genetics, bioactive molecules, fresh and dry matter, type of solvents and conditions during extractions, experimental conditions, etc.). Batista et al. [43] revealed that, when estimating the amount of total phenols in spirulina-enriched cookies, the addition of spirulina at 2% produced no significant difference compared to the control sample, with the amount of phenols being 1.4 mg GAE/g. However, when the spirulina content was increased to 6%, the phenol content increased significantly to 2 mg GAE/g. Jerez-Martel et al. [44] reported that, even when using a concentration of spirulina extract exceeding 40 mg/mL, no noticeable effects on DPPH free radical scavenging were observed.

### 3.2. Chemical Composition of Camel Sausages

Regarding the chemical composition of the freshly made camel sausages, the average results indicated a moisture content of 56.92 ± 1.80%, protein 17.62 ± 0.57%, and fat 11.37 ± 0.42%. This chemical composition is in line with the results from a previous meat sausage study [45] that found 51.3% moisture, 15.9% protein, and 13% fat in sausage made with 100% camel meat.

Unlike traditional types of red meat, camel meat is notably rich in amino acids and minerals and contains a high amount of unsaturated fats. Furthermore, it exhibits hypoallergenic properties, positioning it as a valuable ingredient for the production of functional foods.

Several authors have described the functional properties of food products enriched with algae [46,47,48]. Agregan et al. [49] reported that the fat content of meat products remained unchanged after the addition of seaweed extracts (0.05%). Despite its relatively low fat content, the inclusion of seaweed in meat products could have positive effects on their fatty acid profile, due to the high content of PUFAs.

#### 3.2.1. pH

**Figure 2:**
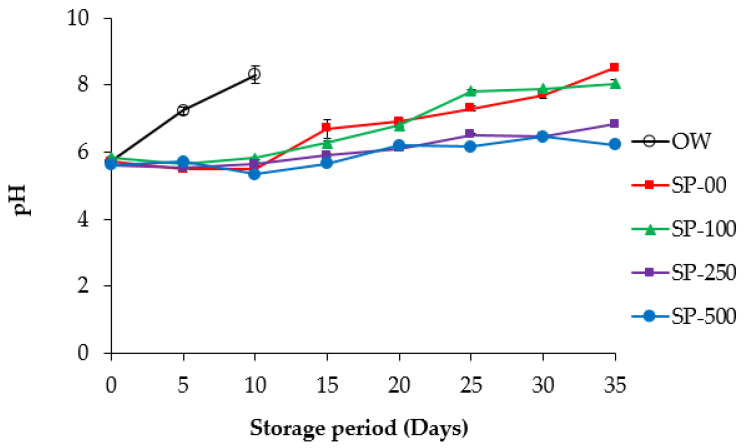
pH values of packed camel sausage stored at 1 ± 1 °C for 35 days. Data are shown as means ± SD. (O) OW: untreated/overwrapped samples packed in air; (■) SP-00: untreated and vacuum-packed samples; (▲) SP-100 samples treated with 100 mg/kg of SP and vacuum-packed; (■) SP-250: samples treated with 250 mg/kg of SP and vacuum-packed; (●) SP-500: samples treated with 500 mg/kg of SP and vacuum-packed. The lack of data after 10 days of storage for the OW samples is due to excessive growth of spoilage-causing microorganisms and a very unpleasant odor.

Figure 2 shows that the initial pH values in all samples were between 5.62 and 5.83. However, a significant increase in pH (*p* < 0.05) was observed in all samples during the storage period, ranging from 6.21 to 8.50 at the end of storage. Nevertheless, it was observed that, during the first two weeks of storage, the groups of samples supplemented with spirulina exhibited relatively stable pH values. This stability could be attributed to the buffering capacity of the meat and/or the antimicrobial activity of the SP, as well as to the spice mixture used, which could inhibit the development of spoilage microbes and, consequently, may have reduced protein degradation in the stored sausages. The intrinsic buffering properties of spirulina should also be considered [50]. The pH of overwrapped sausages stored under aerobic conditions (OW) exhibited a more rapid increase in pH during storage, reaching a value of 8.31 by the tenth day, while the pH of the SP-250- and SP-500-treated samples reached 6.82 and 6.21, respectively, on day 35 of storage. This sharp increase in pH during aerobic storage may be attributed to the release of metabolites from protein degradation, especially basic amines, resulting from microbial activity [51].

Regarding the increase in the pH values of the spirulina-treated samples, similar results were reported by Siladji et al. [47], who noticed that treatment of meat products with algae resulted in a significant increase in pH values over time. This effect varied depending on the concentration and type of algae, as well as the initial pH and the storage time. Similar pH results were also reported by Djenane et al. [19] in modified atmosphere-packed rabbit meat. Several authors suggest that the microbiota present on the meat surface at the beginning of storage can produce and release metabolites from protein degradation, primarily basic amines, which can modify the pH of the stored product [52,53]. To support this hypothesis, Yehia et al. [54] reported a significant decrease in the pH of vacuum-packed camel meat samples treated with chitosan and Citrox, which could be attributed to the antimicrobial activity of both compounds. The pH of algae itself can vary from slightly acidic to slightly alkaline, depending on its composition [55], so its incorporation could affect the pH of meat products. The addition of algal extracts containing 0.5% polysaccharide to Chinese-style sausages reduced the pH during storage by less than 0.1 units [56]. In the present study, the pH values of camel sausage were unaffected by the inclusion of spirulina at any of the three concentrations tested. Voloschenko et al. [57] reported similar results in pork liver pâté supplemented with 2.5% spirulina.

#### 3.2.2. Lipid Peroxidation (TBARS)

In this study, the TBARS assay was used to measure the oxidative stability of camel sausages during storage. The results revealed a steady increase during storage, but with significant differences (*p* < 0.05) between groups. In general, elevated TBARS values indicate poor oxidative stability of the stored product. The TBARS assay is one of the most widely used techniques for the evaluation of secondary oxidation products in animal-derived foods, mainly malondialdehyde (MDA), which is considered to be a key factor in oxidative rancidity and a primary contributor to off flavors in packaged meats [58]. Table 3 shows the results of TBARS values in camel sausages treated with different concentrations of SP during vacuum-chilling storage at 1 ± 1 °C for 35 days. The TBARS results showed a progressive trend in all groups, but a more rapid increase was observed in the OW and SP-00 groups, and the samples from the SP-100 group showed significantly higher values than those recorded in the SP-250 and SP-500 groups.

The lower increase observed in TBARS values with the intermediate (SP-250) and higher (SP-500) spirulina treatments leads us to believe that some components of spirulina may contribute to reducing lipid peroxidation in the stored samples. This effect could be attributed to the presence of polyphenols in SP, which can effectively prevent lipid oxidation by providing hydrogen atoms to free radicals. However, in the lower treatment group (SP-100), TBARS values were significantly higher (*p* < 0.05), which would indicate a threshold value below which spirulina is not able to effectively reduce oxidation. Inhibition of lipid oxidation and hydrogen-donating ability is enhanced by increasing the number of hydroxyl groups in polyphenols.

Alavi and Golmakani [59] reported similar results in stored products with different concentrations of SP, which was more effective than other tested antioxidants. Previous studies pointed out the antioxidant effect of spirulina extracts on different seafood on the basis of the presence of a wide range of bioactive molecules (i.e., flavonoids, phycocyanins, polyphenols) [31,60]. The reduced increase observed in TBARS at higher spirulina treatment aligns with the antioxidative properties of spirulina, as reported by Voloschenko et al. [57], who found that spirulina-enriched pork liver pâtés (1%) had notably lower peroxide values compared to the control, directly proportional to the spirulina concentration. Luo et al. [56] examined the antioxidant effect of spirulina at 0.1%, 0.25%, and 0.5% in Chinese-style sausages. Samples containing spirulina had higher DPPH radical scavenging activity and lower TBARS values during 24 days of storage at 4 °C compared to control samples, and the antioxidant effect was dose-dependent.

The adapted feeding approach and appropriate preservation of the antioxidant status of animals seems to be the most efficient method to preserve the quality of animal products. In this context, spirulina has a demonstrated ability to maintain the activity of some cellular antioxidant enzymes and increase the levels of reduced glutathione (GSH) in cells [41]. Similarly, the addition of seaweed extract (0.02%) extended the shelf life of mechanically separated turkey meat sausages due to reduced TBARS values during refrigerated storage for 15 days [61]. Moroney et al. [62] found that cooked pork patties containing seaweed extract (0.5%) exhibited lower lipid oxidation after 14 days of refrigerated storage compared to untreated samples. In addition, chicken sausages enriched with 2% of three types of seaweed showed significantly lower TBARS values after 28 days of storage [63].

The data shown in Table 3 reveal the progression of TBARS values in the SP-00 group, with 0.51 and 2.92 mg MDA/kg at 0 and 35 days of storage, respectively. However, in the OW samples, TBARS values increased from 0.54 mg MDA/kg at day 0 of storage to 3.82 mg MDA/kg as early as day 10 of storage. The TBARS values of the SP-100 group increased from 0.58 on day 0 of storage to 2.89 mg MDA/kg on day 35. In samples treated with 250 mg/kg SP, TBARS values increased from 0.60 on day 0 of storage to 1.87 mg MDA/kg on day 35. Finally, samples treated with higher concentrations of SP (500 mg/kg) reached lower TBARS values, especially on days 25, 30, and 35 of storage, compared to the SP-00 control group. Sensory evaluation revealed that rancidity in the OW samples developed as early as the fifth day of storage, while the SP-500 samples maintained a normal taste until the end of storage, without developing any rancidity. These results are in agreement with those of Cabrol et al. [48], who reported that frankfurter-type sausages containing 3% algae showed a significant reduction in rancid odor and flavor throughout the refrigerated storage period.

The antioxidant bioactive molecules present in SP can improve the shelf life of food products by delaying lipid oxidation [12,59]. Consequently, spirulina has been extensively investigated as a natural ingredient to improve the quality and stability of meat products [61,63]. As demonstrated in the current study, lipid oxidation increased significantly (*p* < 0.05), reaching 1.89 mg MDA/kg at 5 days of aerobic storage in the OW group. However, when these samples were packaged under vacuum conditions (SP-00), lipid oxidation was significantly reduced and showed 58.73% lower TBARS values (1.89 vs. 0.78 mg MDA/kg) over the same storage period (5 days) (*p* < 0.05). In the SP-added and vacuum-packed groups, the samples further resisted lipid oxidation: in the SP-100, SP-250, and SP-500 groups, lipid oxidation was reduced by 57.67%, 63.49%, and 58.20%, respectively, compared to the OW group after 5 days of storage (*p* < 0.05).

Comparable results were reported by Shafiei and Mostaghim [50], who observed similar trends in TBARS values in calf fillets packaged with a chitosan/natamycin and spirulina-based edible coating, which extended shelf life up to 28 days under refrigerated storage at 4 °C. Soltanizadeh et al. [45] found that TBARS values in vacuum-packed sausages increased gradually due to lipid oxidation over 15, 30, and 45 days of storage. Surprisingly, these authors indicated that TBARS values remained within a low range (0.24 to 0.50 mg MDA/kg), well below the threshold value of 1.5 mg MDA/kg for rancid odor detection [64].

#### 3.2.3. Instrumental Color Parameters

The results of the color parameters of camel sausages during storage are presented in Table 4. An initial observation is that the starting values of lightness (L*), red/green coordinate (a*), and yellow/blue coordinate (b*) are characteristic of high-quality fresh sausages. At the beginning of storage, the OW and SP-00 samples, probably due to the absence of SP in the formulation, exhibited the highest (*p* < 0.05) initial levels of lightness (51.08 and 50.35, respectively), followed by SP-100, SP-250, and SP-500.

During storage, the L* and b* values remained relatively constant (*p* > 0.05); however, the a* value decreased (*p* < 0.05) at the end of storage, probably due to accumulation of metmyoglobin (MetMb), the oxidized form of the oxygen-carrying hemeprotein myoglobin, as a result of pigment oxidation. Similar to our results, Triki et al. [65] observed a pronounced decrease in red color during refrigerated storage of fresh *merguez* sausages. The red color of stored fresh meat is a major aspect for consumers shopping from supermarket display cases and originates when meat myoglobin (Mb) is exposed to oxygen (O_2_), leading to the formation of bright red oxymyoglobin (MbO_2_) [66]. However, in the present study, the loss of a* was less pronounced in sausages treated with higher amounts of spirulina. This natural ingredient appears to stabilize the color of camel sausages and mitigate discoloration, due to its antioxidant activity.

The sensory panel in this study classified the color of camel sausages as less fresh as the storage period advanced and as TPC, TVB-N, and TBARS accumulated. Similar to our results, Frank et al. [67] reported that the surface color of beef was perceived as less fresh by a sensory panel as the aging period progressed and TVB-N accumulated. In addition, Holman et al. [68] suggested that beef color stability is associated with TVB-N concentration, and any decrease in red color is an indicator of blemish and consumer dissatisfaction. A better example was provided by Holman et al. [69], in their global survey of nearly 2800 beef consumers, who reported that beef with a redness score (a* value) below 14.5 was considered unacceptable or spoiled.

The correlation between microbiological and chemical parameters (pH, TPB, and TVB-N) and color parameters was examined and confirmed as an indicator of pork freshness [70]. In this study, the highest a* values were observed in the SP-250 and SP-500 groups throughout the storage period. Camel sausage manufacturing processes involve grinding to reduce particle size, mixing to incorporate spices, and stuffing into casings. During this process, fat smearing may occur, which must be considered, as it affects both the visual and the instrumental quality of the sausage color. In addition, the reduction in redness of camel sausages during storage can be attributed to the conversion of red MbO_2_ to MetMb, a pigment responsible for the development of a brown color [71]. The reduction of MetMb in these products requires either vacuum packaging or injection of O_2_ at saturation levels (70–80%) into the packaging atmosphere [72]. However, the use of high O_2_ systems can accelerate lipid oxidation, leading to MetMb formation. Lagerstedt et al. [73] also found that a higher O_2_ atmosphere is not recommended for beefsteaks in retail tray systems.

These results are in agreement with those of Yehia et al. [54], who reported that citric, malic, and ascorbic acids effectively stabilized lightness and redness while significantly decreased yellowness. Spirulina, due to its high pigment content, can influence the color of products to which it is added. In addition, the use of spirulina at higher concentrations can affect the color of meat products. Camel sausages exhibited high initial a* values (approximately 17), unlike what was observed by Soltanizadeh et al. [45], who found lower values around 13.9. Several authors have established a relationship between redness of meat and fat oxidation [74,75], with higher oxidation levels leading to a decrease in redness. Decreased redness in camel meat generally indicates oxidation of fats and pigments. Some authors have pointed out that the high values of certain color parameters are probably due to the existence of coloring compounds in the spice mixture used for sausage making. These compounds may have increased the redness and yellow color of the sausage samples. A similar observation was reported by our research group for whole rabbit carcasses marinated and packed in a micro-atmosphere with the addition of *ras el-hanout* spice blend [19].

#### 3.2.4. Total Volatile Basic Nitrogen (TVB-N)

**Figure 3:**
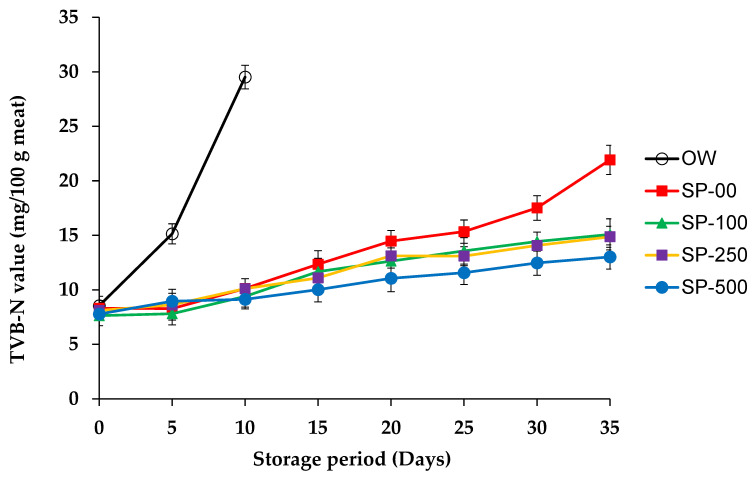
TVB-N values of camel sausages stored at 1 ± 1 °C for 35 days. Data are shown as means ± SD. (O) OW: untreated/overwrapped samples packed in air; (■) SP-00: untreated and vacuum-packed samples; (▲) SP-100: samples treated with 100 mg/kg of SP and vacuum-packed; (■) SP-250: samples treated with 250 mg/kg of SP and vacuum-packed; (●) SP-500: samples treated with 500 mg/kg of SP and vacuum-packed. The lack of data after 10 days of storage for OW samples is due to excessive growth of spoilage-causing microorganisms and a very unpleasant odor.

TVB-N is considered as a reliable indicator for assessing the quality of foods of animal origin, serving as a biomarker of freshness in stored products, especially meat and fish. Higher TVB-N values indicate advanced spoilage, while lower levels indicate freshness [19]. In our study, the chemical stability of camel sausage was significantly affected (*p* < 0.05) by SP treatments and storage conditions. As depicted in Figure 3, TVB-N values significantly increased (*p* < 0.05) during the first days of storage, especially for the OW group consisting of overwrapped sausages stored under aerobic conditions. In this group, TVB-N values increased from 8.54 to 29.52 mg/100 g (3.5 times) within an additional 10 days of storage, taking into account that values exceeding 15–20 mg/100 g are indicative of spoilage. However, the SP-00 group showed a moderate increase in TVB-N, reaching 10.12 mg/100 g over the same period. A comparison of the camel sausage groups revealed that TVB-N values exceeded 15 mg/100 g after just 5 days under aerobic storage conditions (OW), whereas values below this limit were detected after 20 days when the samples were vacuum-packed (SP-00). Indeed, this progression of TVB-N values corresponded closely with the sensory odor analysis that led to the samples being classified as spoiled by the panelists after 5 days of aerobic storage, compared to 20 days for vacuum-packed samples (SP-00). In agreement with our results, Mansur et al. [76] found TVB-N values above 20 mg/100 g after nine days of storage in air and values below this limit after 21 days when beef steaks were vacuum-packed.

The literature contains numerous recommendations regarding TVB-N levels to measure the freshness of meat products. Both the Egyptian Organisation for Standardisation (EOS 1090/2005 [77]) and Quality Control and the Korean Ministry of Agriculture and Forestry [78] advise an upper limit of 20 mg TVB-N/100 g for fresh meat products. However, the Chinese National Food Safety Standard GB2707-2016 [79] sets a limit of 15 mg TVB-N per 100 g. Some authors have proposed acceptable limits of up to 40 mg TVB-N/100 g, although these authors did not provide a reference for this statement. In a recent study, Djenane et al. [19] recommended a value of 20 mg TVB-N/100 g as the upper limit for the initiation of spoilage in modified atmosphere-packed whole rabbit meat. This recommendation was based on microbial spoilage, oxidative reactions, and overall acceptability. Comparable results have been reported, in which TVB-N was positively correlated with the storage period, and which strongly indicates continuous spoilage during storage [80]. In addition, it has often been found that the pH rises during storage and is positively correlated with the TVB-N [19]. This is because meats with a higher pH have lower glycogen or glucose reserves, making proteins and amino acids the primary energy sources for spoilage microorganisms. Consequently, a higher pH promotes proteolysis by these microorganisms, and therefore the accumulation of contaminating nitrogenous proteins.

In the present study, TVB-N levels did not exceed 15 mg/100 g in samples with the highest concentrations of spirulina, while levels reached 21.90 mg/100 g in the SP-00 samples, where spoilage was organoleptically evident. In the SP-100, SP-250, and SP-500 groups, TVB-N values increased from their initial values (day 0) of 7.63, 8.11, and 7.88 mg/100 g, respectively, to 15.09, 14.87, and 13.02 mg/100 mg, respectively, on day 35 of vacuum storage at 1 °C. These results indicate that the addition of spirulina slowed protein breakdown. Compared to an earlier report on rabbit meat, a lower TVB-N index during storage was found in this study. In summary, based on recognized limits stipulating that TVB-N levels should not exceed 15 mg/100 g, the control groups (OW and SP-00) exceeded this limit at 5 and 25 days of storage, respectively, and became unfit for consumption. In contrast, sausages treated with a higher concentration of spirulina (SP-250 and SP-500) remained with acceptable limits until 35 days of storage. This suggests that an upper limit for TVB-N in camel sausages of 15 mg/100 g was established using sensory and microbial spoilage data. Likewise, Holman et al. [68] reported a positive association among TVB-N values and microbial growth in vacuum-packaged beef throughout storage and concluded that values of TVB-N around 15 mg/100 g are indicative of the freshness of the stored meat. In contrast to our results, Luo et al. [81] did not report a decrease in TVB-N in pork sausages treated with spirulina extract compared to control samples.

In our study, increasing the spirulina concentration to 500 mg/kg was more effective in reducing the TVB-N values compared to lower SP concentrations from the 10th day until the end of storage. By 25 days of storage, the TVB-N value of the SP-00 group reached 15.34 mg/100 g, which corresponded to a distinctly unpleasant odor, exceeding the indicative threshold for spoilage. In the SP-100 group, the threshold value of 15 mg/100 g was reached at 35 days. Nevertheless, samples treated with higher spirulina concentrations (groups SP-250 and SP-500) maintained TVB-N values below the limit of 15 mg/100 g throughout the entire storage period. However, the OW samples reached 15.14 mg after only 5 days of storage. The storage atmosphere inside the package is a vital factor in prolonging the shelf life of fresh meat products. In this study, the TVB-N value served as an effective biomarker showing that the addition of spirulina in combination with vacuum packaging significantly inhibited the formation of degrading nitrogen compounds (*p* < 0.05). The TVB-N value increased significantly during storage, and the rate of increase was faster in control samples than in spirulina-enriched samples, which correlated significantly with the sensory quality of the product.

Our results for TVB-N agree with those of Luo et al. [56], who found that TVB-N values and counts of psychrotrophic microorganisms increased in Chinese sausages during 24 days of storage at 4 °C. However, the extent of these changes was also reduced by the addition of spirulina compared to untreated samples. Similarly, our results are comparable to those of Shafiei and Mostaghim [50], who observed a significant decrease in TVB-N values in calf fillets refrigerated for 28 days when coated with an edible package containing spirulina. Comparable results have been reported, in which TVB-N was positively correlated with the storage period, which strongly indicates continuous spoilage during storage [80]. The compounds responsible for this effect may be found in some of the polysaccharides of spirulina or more likely in its bioactive components of a polyphenolic nature. For example, polyphenols from cherry powder have been shown to reduce TVB-N concentrations in meat products under chilling storage [82]. This phenomenon can be explained by the inhibitory effect of polyphenols on endogenous proteases and microbial growth, both of which are ultimately contributors to spoilage in stored meat products [83].

#### 3.2.5. Cooking Characteristics and Shrinkage Measurements

The cooking characteristics (cooking loss, reduction in length, width, and shrinkage) of the camel sausages with added SP as a natural ingredient are shown in Table 5. The sausages formulated with the highest level of SP (250 and 500 mg/kg) exhibited the least reduction in width, length, and shrinkage. Cooking loss refers to the decrease in volume or weight of meat products that occurs throughout the cooking process and can affect the product’s nutritional content, flavor, texture, and color. Thus, the more water a product contains, the brighter it is, as water loss can lead to browning [19]. From our results, it can be deduced that cooking loss increased for all samples as vacuum storage time progressed. In concordance with our results, Triki et al. [65] reported that cooking loss of *merguez* sausages increased during refrigerated storage.

In addition, compared to the vacuum-stored control samples (SP-00), the OW samples showed higher cooking loss during the entire exposure period (10 days). This could be attributed to accelerated proteolysis under aerobic conditions caused by microbial activity, as well as to oxidative reactions occurring during storage. The results also indicated a significant effect (*p* < 0.05) of spirulina addition level on cooking losses. It was observed that formulations with reduced spirulina content (SP-100) experienced higher cooking loss (*p* < 0.05) compared to those in the SP-250 and SP-500 groups, although no significant differences were noticed between the SP-00 control group and the SP-100 group. In general, the highest cooking losses occurred during the initial days of storage. Other authors have observed a reduction in cooking losses in breakfast sausages made with 3% algae [84]. When incorporating three different types of algae at 2–6% into chicken sausages, the decrease in cooking loss was directly proportional to the concentration of algae used [63]. Mohammed et al. [85] studied the effect of incorporating various types of algae into fresh pork sausages and concluded that the addition of 2.5% improved the water-holding capacity (WHC) of the samples without affecting cooking loss. This effect could be attributed to the fact that algae contain fibers which acts as a barrier against water loss, and polysaccharides, which confer gelling ability [86]. Not surprisingly, the inclusion of 2% spirulina significantly increased (54%) the WHC of pâté [57].

Our results could be explained by the influence of spirulina on two key factors, pH value and oxidation, which increase during storage. It is well established that the WHC of meat decreases with pH. Our results suggest the usefulness of spirulina in stabilizing the pH increase and thus improving the WHC of stored samples. This improvement may also be associated with the antioxidant activity of SP bioactive components, which protect the structural integrity of muscle fiber membranes, increasing their capacity to retain water. Supporting this interpretation, Widati et al. [55] demonstrated that the addition of seaweed flour (5%) significantly increased WHC and reduced cooking loss in Indonesian-style beef meatballs compared to control samples without seaweed. A similar effect on WHC was achieved with the addition of chickpea flour [87]. Another study that also used seaweed as an ingredient (6%) in chicken sausages made from mechanically deboned meat reported a significant reduction in cooking loss [88]. In contrast, Marti-Quijal et al. [89] reported that replacing soy protein in fresh pork sausages with seaweed protein did not affect WHC or cooking losses.

During cooking, protein denaturation, water evaporation, loss of meat liquids, and melted fat cause camel sausage to shrink. The shrinkage of camel sausage without SP was greater than that of camel sausage enriched with spirulina. Increasing the proportion of added SP improved the width and length of camel sausages during the cooking process. The reduced shrinkage observed in SP-formulated camel sausages could be due to the binding aptitude of SP, which maintains the functional properties of protein and enhances its ability to retain moisture and melted fat during the cooking process. This phenomenon of reduced shrinkage in cooked meat products has been observed by other authors with the addition of functional ingredients such as pitted olive cake [90], date fruit pulp [91], and Argel leaf powder [92].

### 3.3. Microbiological Quality Evolution 

Due to its particular composition, fresh meat is highly prone to spoilage driven by microbial proliferation and endogenous chemical and biochemical activities, leading to deterioration of its organoleptic properties and subsequent consumer rejection. 

**Figure 4:**
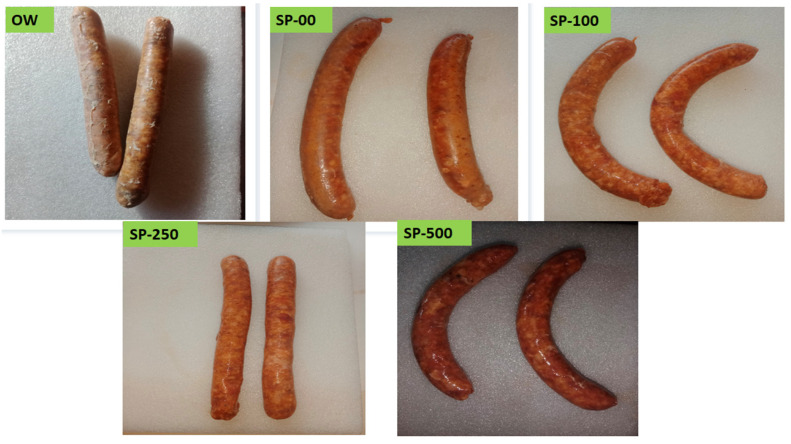
Images of the camel sausages samples fortified with spirulina at 0 to 250 mg/kg (OW: negative control samples after 10 d of aerobic storage; SP-00: sausage with 0 mg/kg of SP after 35 d of vacuum storage; SP-100: sausage with 100 mg/kg of SP after 35 d of vacuum storage; SP-250: sausage with 250 mg/kg of SP after 35 d of vacuum storage; SP-500: sausage with 500 mg/kg of SP after 35 d of vacuum storage.

**Figure 5:**
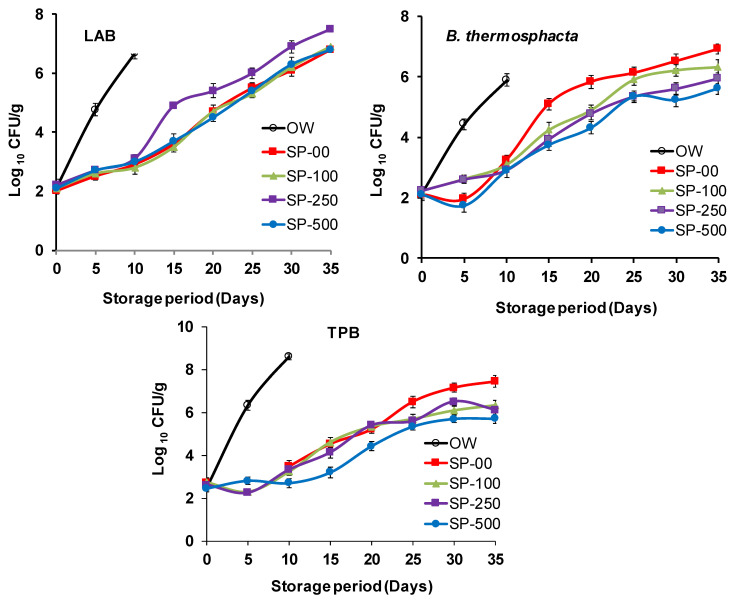
Lactic acid bacteria (LAB), *Brochothrix thermosphacta,* and total psychrotrophic bacteria (TPB) numbers in packed camel sausage stored at 1 ± 1 °C for 35 days. Data are shown as means ± SD. (O) OW: untreated/overwrapped samples packed in air; (■) SP-00: untreated and vacuum-packed samples; (▲) SP-100: samples treated with 100 ppm of SP and vacuum-packed; (■) SP-250: samples treated with 250 ppm of SP and vacuum-packed; (●) SP-500: samples treated with 500 ppm of SP and vacuum-packed. The lack of data after 10 days of storage for the OW samples is due to excessive growth of spoilage-causing microorganisms and a very unpleasant odor.

Figure 4 shows some images of camel sausage samples enriched with spirulina at 0 to 250 mg/kg during storage. The primary bacteria associated with the spoilage of fresh refrigerated meat include psychrotrophic *Pseudomonas* spp., *Brochothrix thermosphacta*, and lactic acid bacteria (LAB). The results presented in Figure 5 illustrate the effect of spirulina supplementation on total psychrotrophic bacteria (TPB), *B. thermosphacta*, and LAB counts over 35 days of storage (*p* < 0.05). Spirulina exhibited a significant impact on TPB and *B. thermosphacta* counts in camel sausage; however, its effect on LAB was not significant, as this bacterial group appears more resilient in the presence of spirulina. Similarly, Beheshtipour et al. [92] studied the effects of *Arthrospira platensis* supplementation on the viability of LAB in foods and found that spirulina stimulates their growth. In our study, the initial TPB levels for the OW, SP-00, SP-100, SP-250, and SP-500 groups were 2.50, 2.72, 2.74, 2.61, and 2.45 log CFU/g, respectively, with no significant differences observed among the groups (*p* > 0.05; Figure 5). This reflects the excellent initial microbiological quality of the raw camel meat, along with the effective maintenance of the cold chain and adherence to hygienic practices during sausage manufacturing. By day 20 of storage, the TPB levels in the SP-100 and SP-250 groups were similar (*p* > 0.05), but significantly lower (*p* < 0.05) than that of the SP-500 group, probably due to the antimicrobial properties of this higher level of spirulina enrichment.

On day 25 of storage, the TPB count of the SP-00 control group was significantly higher (*p* < 0.05) than that of all spirulina-treated groups. A similar situation was observed on days 30 and 35 of storage, reaching 7 log CFU/g, which corresponds to the microbiological limit established by the International Commission on Microbiological Specifications for Foods [93]. This result agrees with that of Deepitha et al. [94], who reported that algal extracts had a beneficial effect on total aerobic count in pangasius fillets until the end of a 20 day refrigerated storage period. Similar to the TPB results, the counts of *B. thermosphacta* from the SP-250 and SP-500 groups were significantly lower (*p* < 0.05) than those from the other groups, and *B. thermosphacta* counts in the SP-100 group were also significantly lower (*p* < 0.05) than those in the OW group up to the 10th day of the storage. *B. thermosphacta* is a facultative anaerobe and, along with pseudomonads (aerobic bacteria), is considered one of the primary spoilage microorganisms specific to stored meat and seafood. The reduced bacterial count can be attributed to the presence of phenolic compounds, which are widely recognized as antibacterial molecules in spirulina (8.94 ± 0.16 mg GAE/g), without underestimating the role that the different bioactive molecules of the spice blend added to the product may play. Several studies have already documented the broad spectrum of antimicrobial effects of spices against various microorganisms [19,75], which is likely to be the case in the present work, all combined with the vacuum cold storage. Meat product packaging under modified atmospheres has made extraordinary progress. The reason for this lies in the ability of this approach to maintain the quality characteristics of these products over a long storage period. Also, the emergence of new pathogenic psychrotrophic bacterial species in packaged meats has prompted researchers to develop preservation barrier systems (Hurdle technology). Biopreservation is the best alternative for meat safety, thanks to the presence of metabolites and bioactive molecules against several pathogenic bacteria [2,19]. Similar to our findings, the antibacterial activity of spirulina has been reported in prior studies. Stejskal et al. [13] demonstrated that hake (*Merluccius merluccius*) fillets packed in crosslinked-gelatine films containing spirulina showed a significant reduction in microbial growth. Alshuniaber et al. [95] and Eid Abdel-Moneim et al. [40] identified phenolic compounds in spirulina such as benzophenone and highlighted their efficacy against gram-positive and gram-negative pathogens. These authors suggested that these compounds in spirulina offer a natural and sustainable basis for future food preservatives.

Furthermore, the antimicrobial activity of spirulina showed a concentration-dependent increase. Our results indicated that spirulina significantly inhibited the growth of TPB and *B. thermosphacta,* and that these effects becoming more pronounced at higher concentrations of spirulina (*p* < 0.05). Consistent with our results, several studies have indicated that spirulina shows broad antimicrobial properties and inhibits mainly pathogenic or spoilage organisms. For example, Metekia and Ulusoy [96] reported that spirulina exerted a large antimicrobial effect on fresh tilapia fish fillets. Bošković Cabrol et al. [48] also reported that frankfurter sausages enriched with 3% microalgae showed lower counts of psychrotrophic bacteria and total viable bacteria by the end of refrigerated storage (60 days). Similarly, in stored pork sausages containing 3% microalgae, a 20% reduction in total aerobic bacteria counts was recorded on day 14 compared to control samples [97]. Microalgae showed a positive influence on the microbiology of beef patties when supplemented at an exceptionally high concentration (>10%), resulting in a significant decrease in total microbiota at the end of refrigerated storage [86].

One study examined the effect of incorporating 0.1 to 0.5% spirulina polysaccharides into Chinese-style sausages on microbiological activity for 24 days at 4 °C. The results showed that the magnitude of growth of mesophilic and psychrotrophic total viable counts was attenuated with spirulina polysaccharides compared to the control group [56]. In another study [50], a spirulina-based edible coating was used for calf fillet refrigerated storage for 28 days. The total microbial load was reduced in all coated samples compared to control samples. Ilieva et al. [98] reported that spirulina exhibited potent antimicrobial activity against bacterial fish pathogens, with MIC values ranging from 2 to 15 μg/mL and an inhibition zone of 50 mm. Righini et al. [99] studied the effects of spirulina compounds against gray mold disease, fungal growth, and spore germination in tomatoes and confirmed their potential as natural compounds for the control of fungal plant pathogens in sustainable agriculture.

### 3.4. Sensorial Quality Evolution

**Figure 6:**
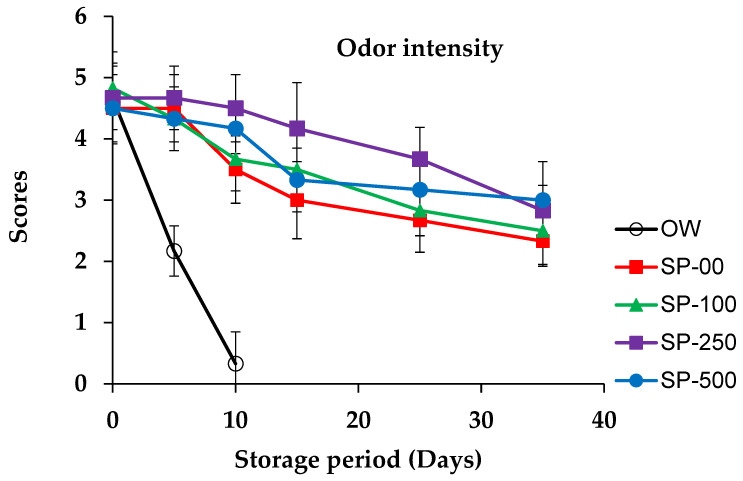
Odor intensity scores of camel sausage stored at 1 ± 1 °C for 35 days. Data are shown as means ± SD. (O) OW: untreated/overwrapped samples packed in air; (■) SP-00: untreated and vacuum-packed samples; (▲) SP-100: samples treated with 100 ppm of SP and vacuum-packed; (■) SP-250: samples treated with 250 ppm of SP and vacuum-packed; (●) SP-500: samples treated with 500 ppm of SP and vacuum-packed. The lack of data after 10 days of storage for the OW samples is due to excessive growth of spoilage-causing microorganisms and a very unpleasant odor.

**Figure 7:**
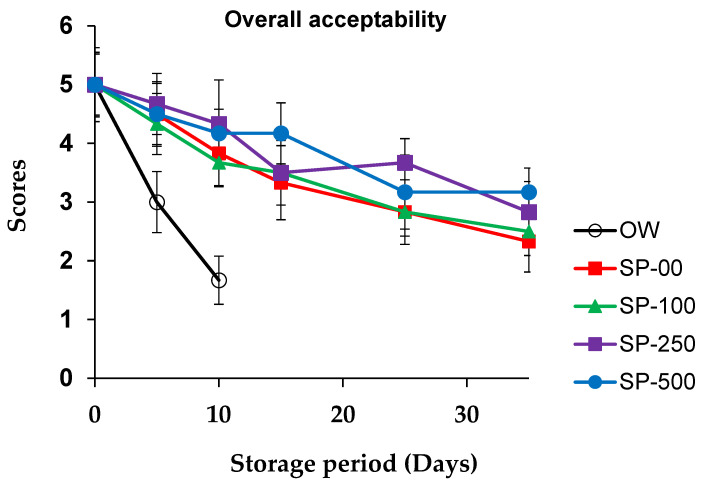
Overall acceptability scores of packed camel *merguez*-type sausage stored at 1 ± 1 °C for 35 days. Data are shown as means ± SD. (O) OW: untreated/overwrapped samples packed in air; (■) SP-00: untreated and vacuum-packed samples; (▲) SP-100: samples treated with 100 ppm of SP and vacuum-packed; (■) SP-250: samples treated with 250 ppm of SP and vacuum-packed; (●) SP-500: samples treated with 500 ppm of SP and vacuum-packed. The lack of data after 10 days of storage for the OW samples is due to excessive growth of spoilage-causing microorganisms and a very unpleasant odor.

The changes in the sensory scores of camel sausage during storage are depicted in Figure 6 and Figure 7. The results of the overall acceptability of the OW camel sausage samples revealed complete spoilage by the 10th day of storage (score = 1.67). Odor intensity strongly affected the product acceptability scale. However, the results revealed that all spirulina-enriched samples exhibited significant (*p* < 0.05) improvement in odor intensity and overall acceptability scores during storage compared to control samples, in a dose-dependent manner. Sensory tests conducted with the group treated with 100 mg/kg SP revealed moderate scores for odor intensity and overall acceptability. On the other hand, spirulina at 250 mg/kg maintained the overall acceptability of sensory properties for more than 25 days, while those treated with 500 mg/kg spirulina (SP-500) maintained overall acceptability of sensory properties even at the end of storage. Yehia et al. [54] found comparable results when *merguez* camel sausage was supplemented with a combination of Citrox and chitosan. In contrast, the control groups (OW and SP-00) showed lower sensory acceptability during storage, with a score falling below 3 even before 10 and 25 days of storage, respectively. Changes in odor intensity during the storage of camel sausages were attributed to both growth of microorganisms and breakdown of their lipid components. It is widely recognized that autooxidation of cell membrane phospholipids is a major contributor to the development of unpleasant odors in stored meat [100].

In general, spirulina has typically been accepted in small amounts when incorporated into food matrices [34], though Han et al. [84] reported that consumers expressed a positive opinion about breakfast sausages containing 1% algae. To avoid potential adverse effects of algae on the sensory acceptability of enriched meat products, Voloschenko et al. [57] recommended limiting the spirulina addition rate to less than 2%. Consequently, in the present study, spirulina powder was used at levels up to 0.05%. Al-Juhaimi et al. [92] reported that the sensory attributes of camel patties were reduced at higher concentrations (4 and 6%) of Algiers leaf powder (ALP) compared to products formulated with 2% ALP or control samples. Similarly, in order not to compromise the flavor of meat sausages, cherry powder should only be added up to 1% [82].

Regarding overall product appearance, our results showed that spirulina maintained the sensory acceptability of treated camel sausages, confirming that spirulina treatment prevents fat oxidation and spoilage bacteria growth, thereby significantly increasing the shelf life of stored camel sausages. Shafiei and Mostaghim [50] investigated the use of a spirulina-based edible coating film on calf fillets during refrigerated storage (28 days) and found that the overall sensory acceptability scores were markedly higher than in the control group. It should also be noted that fat may smear or separate during the manufacture of camel sausages, which may affect consumer visual acceptability [101].

### 3.5. Prebiotic Effect of Spirulina on the Antagonistic Activity of Probiotic Strains Against Pathogenic Bacteria

To ensure high quality and safety of the meat, modified atmosphere packaging (MAP), vacuum packaging (VP), active packaging, intelligent packaging, and so on have been successfully employed, occasionally in combination with natural preservatives [26,102]. The development of camel sausages in the present study required knowledge of the shelf life and behavior of the product during storage, as this type of food is microbiologically perishable and poses potential health risk to consumers. In the arid regions of Algeria, improper temperature management during meat handling is common, requiring additional measures of safety for these products. To the best of our knowledge, the available scientific literature on the microbial safety of camel products remains limited, especially with regard to pathogenic microorganisms. This knowledge gap motivated us to investigate the prebiotic effect of spirulina enrichment on the antimicrobial activity of lactic acid bacteria against pathogenic microorganisms.

**Figure 8:**
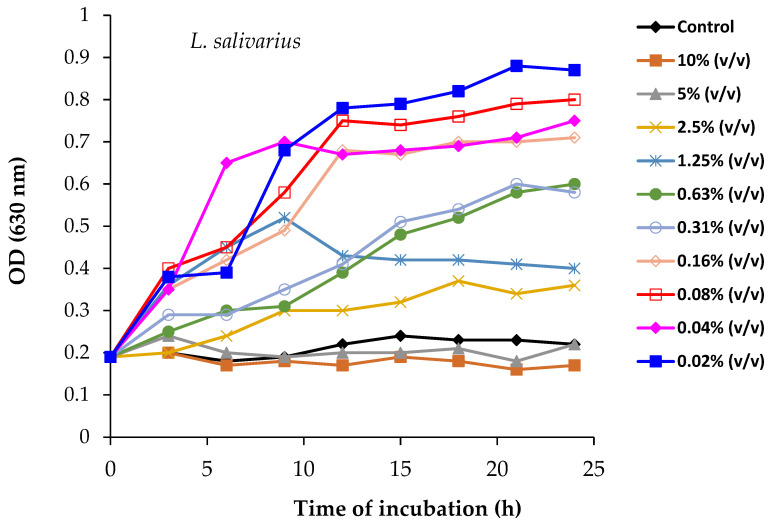
Growth curves of *L. salivarius* in the presence of serial dilutions of SP (10–0.02% *v*/*v*).

**Figure 9:**
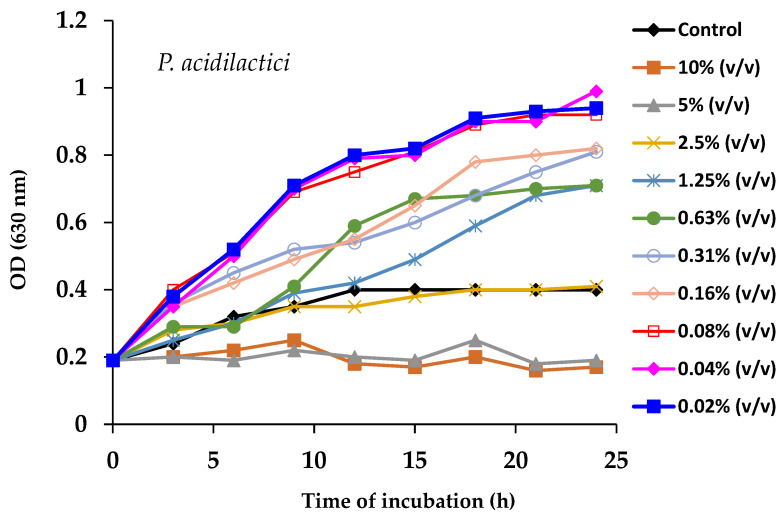
Growth curves of *P. acidilactici* in the presence of serial dilutions of SP (10–0.02% *v*/*v*).

Our in vitro results with *L. salivarius* showed that, while spirulina concentrations of 5% and higher had an inhibitory effect on growth, treatment with concentrations equal to or lower than 0.16% significantly promoted bacterial growth (Figure 8). The intermediate concentrations tested exhibited no marked effect on the growth of *L. salivarius*. Similarly, for *P. acidilactici*, spirulina was toxic at concentrations above 5%, whereas concentrations between 0.02 and 0.08% stimulated bacterial growth. However, intermediate addition levels had no noticeable effect on growth (Figure 9).

Thus, our results clearly illustrate the prebiotic effect of low spirulina concentrations on the growth enhancement of *Lactobacillus salivarius* and *Pediococcus acidilactici*. This observation agrees with previous studies that showed the ability of spirulina to increase the counts of LAB and probiotic strains [103,104]. The cyanobacterium spirulina contains several bioactive components with prebiotic effect that induce the growth of lactic acid bacteria, such as free amino acids, linoleic acid, and adenine [105,106,107]. Gupta et al. [105] investigated the effect of blue-green algae on probiotic bacteria, while subsequent research by Pandey et al. [108] and Lv et al. [109] reported that the use of a combination of prebiotics and probiotics, in the form of a symbiotic, has an additional positive effect compared to their individual use. The beneficial effect of spirulina in promoting the viability and growth of LAB and probiotic bacteria in fermented milk and yogurt has been reported in several studies [103,104]. For example, in a recent study, Rose et al. [28] studied the prebiotic effects of incorporating 0.25% spirulina into dairy products.

**Figure 10:**
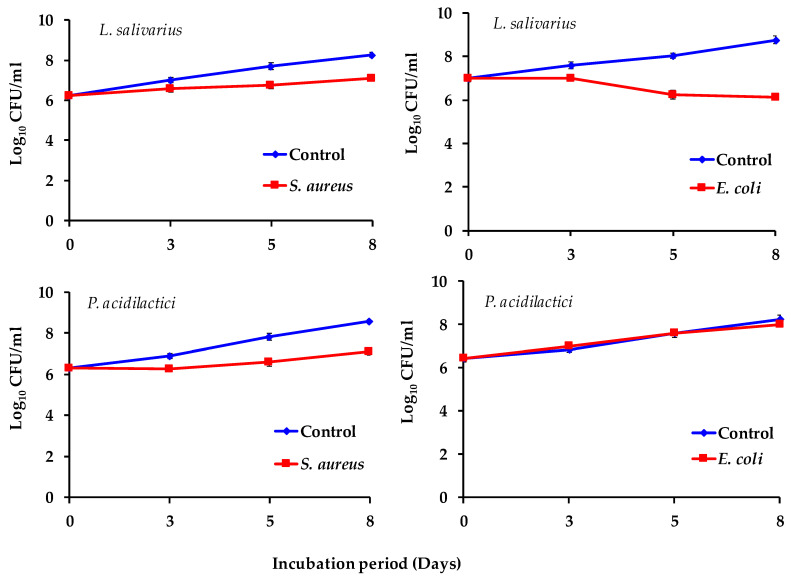
Effects of *L. salivarius* and *P. acidilactici* probiotic strains on the numbers of *S. aureus* and *E. coli* in camel sausage broths with SP treatment, incubated for up to 8 days at 8 ± 2 °C.

The prebiotic effect of spirulina on *L. salivarius* and *P. acidilactici* enhanced their antimicrobial effect against two common foodborne pathogens, *Escherichia coli* O157:H7 and *Staphylococcus aureus*, well-known contaminants of meat and its derivatives. The experiments were carried out in camel sausage broth at a temperature of 8 ± 2 °C to simulate possible temperature abuse along the meat marketing chain. The results (Figure 10) revealed that *E. coli* and *S. aureus* counts obtained from the broth of camel sausage inoculated with *L. salivarius* and *P. acidilactici* were lower than those recovered from control broths without probiotic strains. Specifically, *L. salivarius* inhibited *E. coli* and *S. aureus* growth by 30% and 14%, respectively, after 8 days of incubation, when *P. acidilactici* reduced them by 3% and 17%, respectively, over the same period. Inoculation with probiotic strains did not significantly alter the pH of the broths, indicating that pH was not a contributing factor in pathogen growth inhibition. We believe that the use of probiotic strains may be less effective in the food matrix than in the broths tested, so further research is necessary to comprehensively understand the effect of probiotics and elucidate their precise mechanisms of action.

Several studies have focused on the in vitro antimicrobial effect of numerous algal extracts against *S. aureus* [110] and *E. coli* [111], showing different efficacy depending on the extraction process and the algal species utilized. Phycocyanin extracted from spirulina has been reported to show in vitro antibacterial activity against *S. aureus* and *E. coli* [39]. *Arthrospira platensis* (spirulina) extract exhibited interesting antibacterial activity towards *Listeria monocytogenes* in salmon tartar, as a bacteriostatic using 0.45% of the extract and bactericidal at 0.90%, which could open prospects for its application as a food preservative [112].

The preservation of fresh *merguez* sausages represents a bacteriological and organoleptic challenge. Their poor microbiological and oxidative stability, combined with consumer demands in terms of safety and quality, has prompted researchers to set up a system to optimize their packaging methods. Vacuum packaging is designed to extend shelf life and improve food safety. Improving the process for preserving fresh *merguez* sausage under vacuum for long periods would enable manufacturers to reach domestic or export markets. In terms of packaging, Algeria lags far behind Japan, the United States, and Europe. However, the future of the Algerian meat industry will undoubtedly lie in the development of new technologies for guaranteeing the sanitary quality of the food offered to consumers and preserving the organoleptic properties of these perishable products. Our study has shown that it is feasible to meet the demand of professionals and consumers, since, after more than 4 weeks of storage, the marketing of fresh *merguez* sausage added with spirulina was considered good. This exploratory study sheds light on this issue and improves the state of knowledge on the subject. As a result, the commercial prospects of fresh *merguez* sausage are particularly good, due to such long shelf lives. The results have also demonstrated the possibility of diversifying the range of camel meat products to adapt to longitudinal changes in distribution and consumption patterns and trends, with the aim of creating greater added value. However, it is important to underline that this study was limited to a single packaging method, and that other methods that could be studied in the future (modified atmosphere packaging, active biodegradable packaging, etc.) could have had a different impact.

From a legislative point of view, spirulina is a foodstuff in the EU and is recognized as a food by the Food and Agriculture Organisation of the United Nations (FAO) [113,114]. The Codex Alimentarius lists spirulina extract as a food additive. Spirulina extracts are also food additives approved for use as food colors in the United States, China, Japan, and Korea. The US Food and Drug Administration (FDA) has assigned Generally Recognized As Safe (GRAS) status to spirulina, at doses of 3–6 g/d [115].

## 4. Conclusions

Fresh camel meat products are an essential part of a balanced diet for the Saharan population, aimed at maintaining good health and well-being. Due to their water and nutrient content, these products are highly perishable, which makes them vulnerable to spoilage by microorganisms, as well as contamination with pathogenic bacteria. In light of lifestyle changes among the Saharan population, and with the aim of promoting consumption of camel meat products in their diet, optimizing manufacturing process to improve the quality, shelf life, and safety of ready-to-eat camel products is essential. In Algeria, camel meat is often used as a raw material in home cooking or sold along Saharan roads, with minimal industrial processing. For this reason, scientifically based recommendations and advances in processing this type of raw material are needed.

In light of the results obtained from this investigation, spirulina powder emerges as an innovative and attractive ingredient that can be successfully incorporated at concentrations between 100 and 500 mg/kg in spiced camel sausages. This enrichment can improve quality and functionality without significantly compromising sensory acceptability. The results indicate that fortification of *merguez* camel sausages with 0.025% and 0.05% spirulina effectively inhibited pigment and fat oxidation and slowed both the growth of spoilage microbiota and the increase of TVB-N during storage. These promising results suggest that the shelf life of spirulina-supplemented and vacuum-packed camel sausages can be extended to 35 days under refrigerated conditions.

Moreover, the microbial evidence from this study suggests that spirulina may exhibit prebiotic ability, in that it promotes the growth of lactic acid bacteria used as probiotics. Therefore, camel sausages with spirulina have potential as a functional food, with the possible beneficial effects of probiotics when ingested through the diet. Further research supported by in vivo studies is needed to comprehensively understand the prebiotic effect of spirulina and its mechanisms of action. Educating consumers about the beneficial properties of these products can be a crucial factor in determining their preferences and the price they are willing to pay.

The present research offers valuable contributions to the creation of innovative products that meet the health and development needs of the populations of southern Algeria and contributes to the sustainable development of the Saharan regions. Furthermore, this article supports the idea of incorporating spirulina into the meat sector, both from a nutritional and a health point of view, given its numerous advantages. As consumer demand for high quality, functional meat products continues to rise, spirulina is positioned to become a vital component in future advances in the meat sector. This study presents a minor limitation that could be addressed in future research. An evaluation of the antibacterial properties of SP against the tested pathogenic bacteria could be carried out by direct inoculation into camel sausages. In this way, we could explain whether camel sausages enriched with spirulina are safe from the point of view of microbiological quality.

## Figures and Tables

**Table 1:** Ingredients (%) of *merguez*-type camel sausage formulated with spirulina powder (SP).

Ingredients (%)	Control OW ^1^ and SP-00	SP-100	SP-250	SP-500
Camel meat	70.00	70.00	70.00	70.00
Camel humped fat	20.00	20.00	20.00	20.00
Whole liquid eggs	5.50	5.49	5.47	5.45
Salt	2.00	2.00	2.00	2.00
Spirulina powder (SP)	0.00	0.01	0.025	0.05
*Merguez* spice mixture:	2.50	2.50	2.50	2.50
-Garlic	0.80	0.80	0.80	0.80
-Hot pepper	0.50	0.50	0.50	0.50
-Paprika	0.30	0.30	0.30	0.30
-Caraway	0.20	0.20	0.20	0.20
-Black pepper	0.20	0.20	0.20	0.20
-Coriander	0.50	0.50	0.50	0.50

^1^ control sausages (without spirulina) were stored overwrapped under aerobic conditions.

**Table 2:** Chemical composition and total phenolic content of the spirulina powder used in this study.

Component	Values Per 100 g
Moisture	4.15 ± 0.05
Protein ^1^	66.88 ± 1.16
Fat	3.13 ± 0.06
Carbohydrates ^2^	20.73 ± 1.38
Ash	5.11 ± 0.13
Energy (kcal) ^3^	378.62 ± 0.41
Energy (Kj) ^4^	1584.16 ± 1.74
Total phenolic content (TPC) by Folin–Ciocalteu	
8.94 ± 0.16 mg GAE/g	

^1^ Kjeldahl-N × 5.95. ^2^ Difference (100 − (g[water + protein + fat + ash] in 100 g). ^3^ Energy value (Kcal) = (%Protein × 4) + (%Fat × 9) + (%Carbohydrate × 4). ^4^ Energy value (KJ) = (%Protein × 17) + (%Fat × 37) + (%Carbohydrate × 17).

**Table 3:** TBARS values of camel sausages treated with different concentrations of spirulina powder during vacuum-chilling storage at 1 ± 1 °C for 35 days (mean ± SD).

	Spirulina Powder Concentrations				
Storage (Days)	OW	SP-00	SP-100	SP-250	SP-500
0	0.54 ± 0.01 aA	0.51 ± 0.01 aA	0.58 ± 0.01 aA	0.60 ± 0.01 aA	0.52 ± 0.01 aA
5	1.89 ± 0.07 bB	0.78 ± 0.05 aA	0.80 ± 0.07 aAB	0.69 ± 0.09 aA	0.79 ± 0.05 aA
10	3.82 ± 0.09 cC	1.11 ± 0.09 bAB	1.21 ± 0.08 bC	0.79 ± 0.07 aA	0.87 ± 0.09 aAB
15	nd * (Decomposed)	1.35 ± 0.10 bB	1.34 ± 0.09 bC	1.04 ± 0.08 aAB	1.01 ± 0.03 aB
20	nd (Decomposed)	1.47 ± 0.09 abB	1.42 ± 0.09 abC	1.22 ± 0.03 aB	1.12 ± 0.07 aB
25	nd (Decomposed)	2.11 ± 0.05 bC	2.09 ± 0.10 bD	1.19 ± 0.06 aB	1.18 ± 0.08 aBC
30	nd (Decomposed)	2.51 ± 0.09 cD	2.43 ± 0.04 cE	1.53 ± 0.07 abC	1.33 ± 0.02 aC
35	nd (Decomposed)	2.92 ± 0.09 cE	2.89 ± 0.07 cF	1.87 ± 0.06 abE	1.46 ± 0.09 aC

Notes: Data are shown as means ± SD. Values followed by different letters (a–c) in a row are significantly different (*p* < 0.05); values followed by different letters (A–F) in a column are significantly different (*p* < 0.05). OW: Untreated overwrapped samples maintained at aerobic conditions. * The lack of data (nd: not determined) after 10 days of storage for the OW samples is due to excessive growth of spoilage-causing microorganisms and a very unpleasant odor. Camel sausages treated with a higher concentration of SP (SP-500) did not spoil even after 35 days of storage.

**Table 4:** Color coordinates (CIE Lab) values of the camel *merguez*-type sausages treated with different concentrations of *Spirulina platensis* powder during vacuum-chilling storage at 1 ± 1 °C for 35 days (mean ± standard deviation).

Parameters	Samples	Storage (Days at 1 °C)							
		0	5	10	15	20	25	30	35
	OW	51.08 ± 1.28 aBC	48.42 ± 2.52 aAB	49.35 ± 2.12 aB	nd *	nd	nd	nd	nd
	SP-00	50.35 ± 2.20 aB	47.54 ± 2.00 aA	49.35 ± 2.12 aB	50.15 ± 2.25 aAB	48.30 ± 1.69 aA	51.25 ± 0.75 aB	48.75 ± 1.15 aB	46.37 ± 0.85 aA
CIE L*	SP-100	49.35 ± 2.12 aB	48.35 ± 1.20 aAB	52.24 ± 0.57 bC	51.25 ± 1.15 abB	49.47 ± 2.09 aA	51.09 ± 2.07 abB	51.09 ± 2.24 abC	48.24 ± 2.35 aAB
	SP-250	47.15 ± 2.25 aA	49.15 ± 3.05 abB	48.31 ± 1.10 aA	49.25 ± 2.08 abA	49.19 ± 0.79 bA	50.15 ± 2.10 bB	50.33 ± 3.13 bC	49.05 ± 2.35 abB
	SP-500	46.38 ± 2.35 aA	46.45 ± 0.85 aA	47.35 ± 2.10 aA	49.05 ± 2.07 abA	47.75 ± 1.15 aA	47.35 ± 2.02 aA	46.85 ± 1.10 aA	47.56 ± 1.17 aA
	OW	18.13 ± 1.17 cAB	13.42 ± 1.42 bA	06.18 ± 0.87 aA	nd	nd	nd	nd	nd
	SP-00	17.58 ± 2.10 eA	18.24 ± 2.23 eBC	15.49 ± 1.15 dB	16.38 ± 1.15 dA	13.21 ± 2.20 cA	10.53 ± 0.50 bA	08.15 ± 1.10 aA	08.09 ± 1.08 aA
CIE a*	SP-100	17.25 ± 1.19 dA	17.18 ± 1.25 dB	15.36 ± 0.19 bcB	15.32 ± 1.20 bcA	14.01 ± 0.35 bA	13.73 ± 1.50 bB	11.35 ± 1.15 aB	10.12 ± 1.05 aB
	SP-250	16.87 ± 0.87 abA	16.68 ± 1.25 abB	15.79 ± 1.18 aB	16.02 ± 1.37 abA	16.09 ± 0.20 abB	15.70 ± 0.58 aC	14.75 ± 1.12 aC	14.09 ± 1.05 aC
	SP-500	17.16 ± 1.21 bA	17.35 ± 2.20 bB	17.29 ± 1.10 bC	16.89 ± 1.20 abAB	16.56 ± 2.14 abB	15.63 ± 1.19 aC	15.15 ± 1.32 aC	14.65 ± 0.85 aC
	OW	23.29 ± 1.35 aB	21.75 ± 2.33 aB	22.85 ± 1.71 aB	nd	nd	nd	nd	nd
	SP-00	23.19 ± 2.10 cdB	23.09 ± 2.37 cdB	23.01 ± 1.40 cdB	21.57 ± 2.13 cB	19.28 ± 2.06 bAB	17.33 ± 2.11 aA	16.29 ± 0.98 aA	17.29 ± 1.48 aAB
CIE b*	SP-100	21.50 ± 2.35 cAB	21.10 ± 095 cAB	19.19 ± 1.65 bcA	18.31 ± 3.08 bA	17.11 ± 1.24 abA	16.84 ± 2.21 aA	17.17 ± 2.27 abAB	15.28 ± 1.74 aA
	SP-250	20.47 ± 2.33 cA	19.49 ± 2.33 cA	20.08 ± 0.87 cA	17.29 ± 1.14 abA	18.42 ± 1.27 abA	16.29 ± 0.98 aA	17.11 ± 1.24 abAB	15.89 ± 2.30 aA
	SP-500	19.59 ± 2.73 cA	21.29 ± 2.07 cdAB	19.28 ± 2.06 cA	20.53 ± 1.34 cB	18.79 ± 2.41 bA	17.19 ± 2.12 abA	15.89 ± 2.07 aA	16.19 ± 2.07 aA

Notes: Data are shown as means ± SD. Values followed by different letters (a–e) in a row are significantly different (*p* < 0.05); values followed by different letters (A–C) in a column are significantly different (*p* < 0.05). OW: Untreated overwrapped samples maintained at aerobic conditions (negative control). * The lack of data (nd: not determined) after 10 days of storage for OW samples is due to excessive growth of spoilage-causing microorganisms and a very unpleasant odor. Camel sausages treated with a higher concentration of SP (SP-500) did not spoil even after 35 days of storage.

**Table 5:** Cooking loss and shrinkage measurements of camel sausages at 1 ± 1 °C over 35 days (mean ± SD).

Samples	Vacuum Storage (Days)			
	0	10	20	35
	Cooking loss %			
OW	32.08 ± 2.18 bD	25.60 ± 0.58 aB	nd *	nd
SP-00	31.11 ± 0.99 abD	32.69 ± 1.48 bD	30.09 ± 2.01 aBC	32.48 ± 0.62 bC
SP-100	29.71 ± 2.19 aC	31.18 ± 0.95 bC	28.84 ± 1.50 aB	31.68 ± 1.66 bC
SP-250	26.48 ± 0.79 aB	26.07 ± 1.86 aB	27.69 ± 2.70 aB	26.87 ± 0.96 aB
SP-500	24.41 ± 1.70 bA	22.55 ± 1.97 aA	23.88 ± 1.68 abA	23.56 ± 1.69 abA
	Reduction in width %			
OW	24.60 ± 1.08 aE	26.49 ± 0.80 bD	nd	nd
SP-00	22.51 ± 0.90 bD	19.60 ± 0.80 aC	22.75 ± 1.25 bBC	25.72 ± 0.55 cBC
SP-100	19.42 ± 0.99 aC	20.16 ± 2.05 aC	21.18 ± 1.30 bB	24.54 ± 0.92 cB
SP-250	17.57 ± 0.72 abB	17.53 ± 1.05 abB	16.66 ± 0.40 aA	15.65 ± 0.71 aA
SP-500	14.65 ± 0.83 aA	13.65 ± 0.53 aA	15.42 ± 0.98 abA	15.06 ± 0.63 abA
	Reduction in length %			
OW	17.71 ± 0.34 aC	17.96 ± 0.31 aD	nd	nd
SP-00	16.72 ± 0.76 aC	17.20 ± 0.29 aD	19.64 ± 0.60 bD	21.72 ± 1.67 cD
SP-100	17.17 ± 0.51 bC	15.60 ± 0.62 aC	16.77 ± 0.98 abC	18.48 ± 1.08 cC
SP-250	14.68 ± 0.61 aB	13.65 ± 0.41 aB	14.76 ± 0.51 aB	15.35 ± 1.01 abB
SP-500	11.42 ± 0.61 abA	10.00 ± 0.28 aA	10.44 ± 1.17 aA	11.25 ± 0.84 abA
	Shrinkage %			
OW	20.17 ± 1.28 aE	21.40 ± 1.25 abD	nd	nd
SP-00	18.96 ± 0.61 aD	20.40 ± 0.62 bD	21.81 ± 0.29 bcD	21.86 ± 0.75 bcB
SP-100	15.99 ± 0.50 aC	17.07 ± 0.18 bC	20.12 ± 0.87 cC	22.26 ± 0.53 dB
SP-250	12.82 ± 0.26 aB	13.36 ± 0.56 aB	14.92 ± 0.93 abB	16.85 ± 0.25 cA
SP-500	10.55 ± 0.55 aA	11.62 ± 0.78 abA	13.35 ± 0.55 cA	16.05 ± 0.40 dA

Notes: Data are shown as means ± SD. Values followed by different letters (a–d) in a row are significantly different (*p* < 0.05); values followed by different letters (A–E) in a column are significantly different (*p* < 0.05). OW: Untreated overwrapped samples maintained at aerobic conditions. * The lack of data (nd: not determined) after 10 days of storage for OW samples is due to excessive growth of spoilage-causing microorganisms and a very unpleasant odor.

## Data Availability

The original contributions presented in the study are included in the article, further inquiries can be directed to the corresponding author.
